# Mitotic slippage in non-cancer cells induced by a microtubule disruptor, disorazole C_1_

**DOI:** 10.1186/1472-6769-10-1

**Published:** 2010-02-11

**Authors:** Fengfeng L Xu, Youssef Rbaibi, Kirill Kiselyov, John S Lazo, Peter Wipf, William S Saunders

**Affiliations:** 1 Department of Biological Sciences, University of Pittsburgh, Pittsburgh, Pennsylvania 15260, USA; 2 Department of Pharmacology and Chemical Biology, University of Pittsburgh School of Medicine, Pittsburgh, Pennsylvania 15261, USA; 3 Department of Chemistry, University of Pittsburgh, Pittsburgh, Pennsylvania 15261, USA

## Abstract

**Background:**

Disorazoles are polyene macrodiolides isolated from a myxobacterium fermentation broth. Disorazole C_1 _was newly synthesized and found to depolymerize microtubules and cause mitotic arrest. Here we examined the cellular responses to disorazole C_1 _in both non-cancer and cancer cells and compared our results to vinblastine and taxol.

**Results:**

In non-cancer cells, disorazole C_1 _induced a prolonged mitotic arrest, followed by mitotic slippage, as confirmed by live cell imaging and cell cycle analysis. This mitotic slippage was associated with cyclin B degradation, but did not require p53. Four assays for apoptosis, including western blotting for poly(ADP-ribose) polymerase cleavage, microscopic analyses for cytochrome C release and annexin V staining, and gel electrophoresis examination for DNA laddering, were conducted and demonstrated little induction of apoptosis in non-cancer cells treated with disorazole C_1_. On the contrary, we observed an activated apoptotic pathway in cancer cells, suggesting that normal and malignant cells respond differently to disorazole C_1_.

**Conclusion:**

Our studies demonstrate that non-cancer cells undergo mitotic slippage in a cyclin B-dependent and p53-independent manner after prolonged mitotic arrest caused by disorazole C_1_. In contrast, cancer cells induce the apoptotic pathway after disorazole C_1 _treatment, indicating a possibly significant therapeutic window for this compound.

## Background

Microtubules are dynamic polymers that facilitate transport and movement within the cell [1]. Microtubule dynamics are a critical aspect of mitosis, ensuring accurate chromosome capture and segregation [1,2]. Factors that interfere with microtubule attachment keep the mitotic spindle checkpoint unsatisfied, thus causing mitotic arrest and inhibition of cell proliferation [3].

Microtubule dynamics can be modified by two groups of chemical inhibitors. The first group, represented by taxanes, stabilizes microtubules and is clinically used to treat breast, lung, bladder and head and neck cancers [4]. The second group of modifiers include vinblastine, vincristine, and vinorelbine, disrupt microtubules and are used in the treatment of leukemia, lymphoma, small cell lung and breast cancer, and other malignancies [5]. Although these chemotherapeutic drugs are efficacious, new drug development is still needed due to intrinsic and acquired drug resistance and untoward actions of existing therapeutics.

Intensive research has focused on the responses of cancer cells to microtubule inhibitors, and it is known that apoptosis is a common outcome triggered by either p53-dependent or p53-independent pathways [[6,7] and references within]. However, the response of non-transformed cells to microtubule inhibitors is less well understood. Contrasting the response of non-cancer cells and tumor cells should enhance our understanding of the effects of microtubule inhibitors on cancer patients.

The disorazoles are a family of natural compounds isolated from the fermentation broth of the myxobacteria *Sorangium cellulosum *[8]. Disorazole A_1_, one of the major components, was demonstrated to disrupt microtubules, leading to mitotic arrest and eventually apoptosis [9]. Disorazole C_1 _(DZ), a relatively minor constituent of the fermentation mixture, was obtained synthetically since it was considered to have greater therapeutic potential due to the absence of the chemically reactive divinyl oxirane and (*E, Z*)-dienyl oxazole moieties [8,10,11] (Additional file 1). DZ was found to cause microtubule depolymerization and mitotic arrest during a small molecule screen [11]. In this paper, we further examined the cellular responses to DZ and compared our results to two known microtubule inhibitors, vinblastine (VBL) and taxol (TXL).

## Results

### 1. DZ has the classical properties of microtubule inhibitors, including mitotic arrest and inhibition of cell proliferation

**Figure 1:**
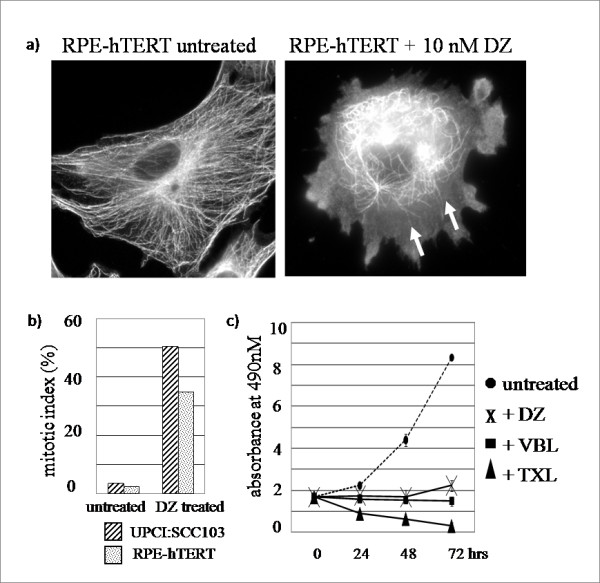
**DZ depolymerizes microtubules from the plus-ends, arrests cells in mitosis and inhibits cell proliferation**. a) Depolymerization of microtubules. RPE-hTERT cells were treated with 10 nM of DZ for 1 hour, fixed and stained with antibody against α-tubulin. Arrows indicate the retracted microtubules. b) Mitotic arrest. UPCI:SCC103 and RPE-hTERT cells were treated with 50 nM of DZ for 16 hours and visualized by DAPI staining. c) Inhibition of cell proliferation as determined by the MTS assay (Promega). HFF-hTERT cells were treated with 10 nM of DZ, 100 nM of VBL or 1 μM of TXL for the indicated times. Results are averages of two experiments.

To analyze the effect of DZ on microtubules, we preformed indirect immunofluorescence on immortalized retinal pigmented epithelial cells (RPE-hTERT) treated with 10 nM of DZ, a concentration similar to DZ's IC_50 _concentrations in several tested cell lines [12]. One hour after DZ was added, microtubules were observed to have retracted from the cell periphery, confirming that DZ depolymerized microtubules and showing that the overall disruption was more severe at the distal plus ends (Figure 1a). Similar results were observed in cancer cells treated with DZ (data not shown).

One property that microtubule inhibitors share is that they cause mitotic arrest by activating or preventing the satisfaction of the spindle assembly checkpoint [3]. DAPI-staining of chromosomes was used to demonstrate an increase in the mitotic index in both non-cancer and cancer cells 16 hours after DZ treatment (Figure 1b). Similar results were observed using phosphorylated histone H3 staining (data not shown).

Finally, cell proliferation is typically blocked by microtubule disruption. After treatment with 10 nM of DZ, the proliferation of immortalized human foreskin fibroblast cells (HFF-hTERT) was inhibited, as determined by the MTS assay (Figure 1c). Similar results were also obtained in RPE-hTERT cells (Additional file 2). In summary, DZ disrupts microtubules, arrests cells in mitosis and inhibits cell proliferation, similar to classical tubulin inhibitors.

### 2. After a prolonged mitotic arrest, cells treated with DZ underwent mitotic slippage

**Figure 2:**
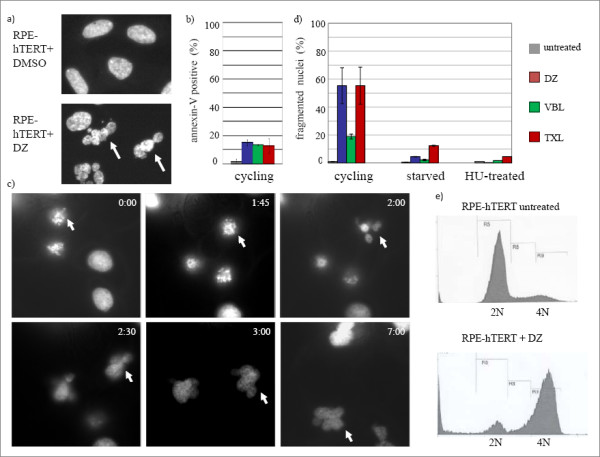
**Mitotic slippage after DZ treatment**. a) Fragmented nuclei (arrows) in RPE-hTERT cells treated with either DMSO as a control or 10 nM of DZ for 38 hours and visualized by DAPI staining. b) Percentage of annexin-V positive cells (apoptotic cells) in untreated RPE-hTERT cells and RPE-hTERT cells treated with 10 nM of DZ, 100 nM of VBL, 1 uM of TXL for 38 hours. c) Live cell imaging of mitotic slippage. UPCI:SCC40 cells stably-transfected with GFP-H2B were pre-treated with 10 nM of DZ for 16 hours before analysis (also see the movie in Additional file 3). Arrow points to a cell that is first in mitosis and then undergoing mitotic slippage resulting in abnormally shaped nuclei. Timestamps: hours:minutes. d) Percentages of the fragmented nuclei (mitotic slippage) in cycling RPE-hTERT cells and RPE-hTERT cells arrested in the different phases of cell cycle. Serum-starvation or 4 mM hydroxyurea (HU) were used to stop cell cycling prior to the treatment with 10 nM of DZ, 100 nM of VBL, 1 μM of TXL for 38 hours. Results were averages of three independent experiments. e) Flow cytometry analysis of DNA content. RPE-hTERT cells were treated with 10 nM of DZ for 38 hours. Data are representative for three independent experiments.

After 38-hour treatment with 10 nM of DZ, many RPE-hTERT cells showed fragmented nuclei (Figure 2a), which were also observed in VBL or TXL treated cells (data not shown). This nuclear fragmentation was not due to apoptosis in these cells as demonstrated by low annexin-V staining (Figure 2b); rather, it was similar to the phenotype observed in cells abnormally exiting from a prolonged mitotic arrest, referred to as mitotic slippage [13]. To test directly whether mitotic slippage was occurring in response to DZ treatment, we exploited live cell imaging. Due to the low transfection efficiency of RPE-hTERT cells, we examined UPCI:SCC40 cells (an oral squamous cell carcinoma cell line, a gift of Dr. Susanne Gollin, University of Pittsburgh) stably-transfected with GFP-tagged histone H2B. After treatment with 10 nM of DZ for 16 hrs, most of the observed mitotic cells exited mitosis to form fragmented nuclei with decondensed chromosomes (Figure 2c, also see the movie in Additional file 3) within the 9.5 hour recording window (note that the arrow in Figure 2c points to the same cell at different time points). Of 23 cells arrested in mitosis, we found that 70% of them (16/23) underwent mitotic slippage, forming fragmented and decondensed micronuclei similar to the example shown in Figure 2c. An additional 13% of cells underwent mitotic slippage and then cell death, which was characterized by chromosome decondensation shortly followed by recondensation and fragmentation of the chromatin into many small pieces. Another 13% of cells underwent a mitotic catastrophe, characterized by many small and highly condensed chromatin fragments formed directly from mitotic arrested cells. Finally, additional cells (~4%) maintained the mitotic arrest; these cells may be entering cellular senescence, seen previously with DZ treatment [12].

To confirm that mitotic slippage was the source of the fragmented nuclei in the majority of the population, serum starvation was used to arrest cells in G1. After a 38-hour treatment with 10 nM of DZ, less than 5% of the serum-starved RPE-hTERT cells showed fragmented nuclei, indicating that the observed nuclear fragmentation required progression through the cell cycle (Figure 2d). Similar results were obtained in hydroxyurea-treated cells, which were arrested in S phase, confirming that the nuclear fragmentation required transit through mitosis (Figure 2d). Although the cycling cells were able to escape the DZ-induced arrest, they did not resume a normal cell cycle but arrested again in G1, as indicated by FACS analysis at 38 hours of DZ exposure (Figure 2e).

**Figure 3:**
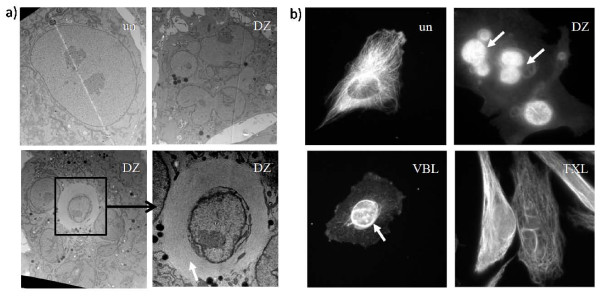
**Microtubule filaments form around nuclei after DZ treatment**. a) Electron microscopy analysis of the nuclei in untreated RPE-hTERT cells and RPE-hTERT cells treated with 10 nM of DZ for 38 hours. A white arrow indicates the microtubule-like structures around the fragmented nucleus in the blowout image. b) Immunofluorescence microscopy analysis of tubulin structures in untreated RPE-hTERT cells and RPE-hTERT cells treated with 10 nM of DZ, 100 nM of VBL, 1 μM of TXL for 38 hours. Arrows indicate the microtubule-like structures around the fragmented nucleus labeled by anti-tubulin antibodies. un: untreated.

Interestingly, upon examination of those fragmented micronuclei, filaments were found to accumulate around them forming a thick bundle, as shown by electron microscopy (Figure 3a). These filaments stain positive with anti-tubulin antibodies by immunofluorescence (Figure 3b) and we believe they are variants of the microtubule structures seen in untreated cells. Similar perinuclear accumulation of microtubule-like structures was seen previously with a vinca alkaloid microtubule inhibitor [14]. The microtubules appear to be forming an abnormal array around the micronuclei, which might contribute to the fragmentation observed after DZ treatment, but this will require further analysis.

### 3. Mitotic slippage in non-cancer cells induced by DZ is correlated with cyclin B degradation

**Figure 4:**
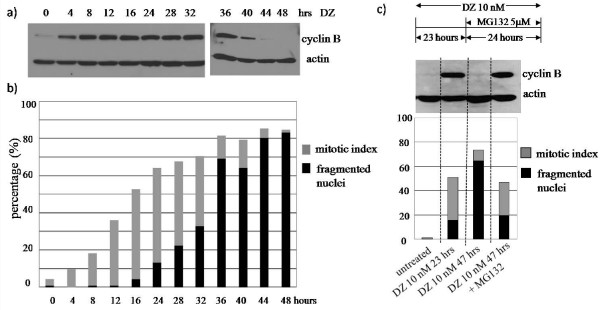
**Cyclin B degradation is correlated with mitotic slippage**. RPE-hTERT cells were treated with 10 nM of DZ in the following experiments. a) Immunoblot analysis of cyclin B during a time course from 4 to 48 hours. Actin was a loading control. b) Percentages of fragmented nuclei analyzed at the same time points as in a. c) Upper panel: a schematic experimental design. Cells were treated with DZ for 47 hours. 5 μM MG132 was added 23 hours after DZ was added to avoid a lethal toxicity effect on the cells. Lower panels: cyclin B protein levels and percentages of fragmented nuclei analyzed as in above experiments. Data are representative for three independent experiments.

Previous studies indicate that mitotic slippage occurs via slow cyclin B degradation and abrogation of the mitotic spindle assembly checkpoint [16]. In our study, the protein level of cyclin B accumulated up to 36 hours in RPE-hTERT cells upon DZ treatment and then sharply decreased (Figure 4a). Similarly, the percentage of cells undergoing mitotic slippage began to peak at 36 hours (Figure 4b), suggesting that cyclin B degradation was also associated with mitotic slippage after DZ treatment.

To study whether protein degradation was required for the observed mitotic slippage, we used the protease inhibitor MG132 to block cyclin B degradation. In MG132-treated cells, the percentage of cells undergoing mitotic slippage was dramatically reduced while the mitotic index remained high, indicating that cells blocked for proteasomal protein degradation were less likely to undergo mitotic slippage from DZ (Figure 4c). Our data support the conclusion that cyclin B degradation was correlated with mitotic slippage in non-cancer cells after DZ treatment.

### 4. p53 is stabilized after DZ treatment, but p53 is not required for mitotic slippage

**Figure 5:**
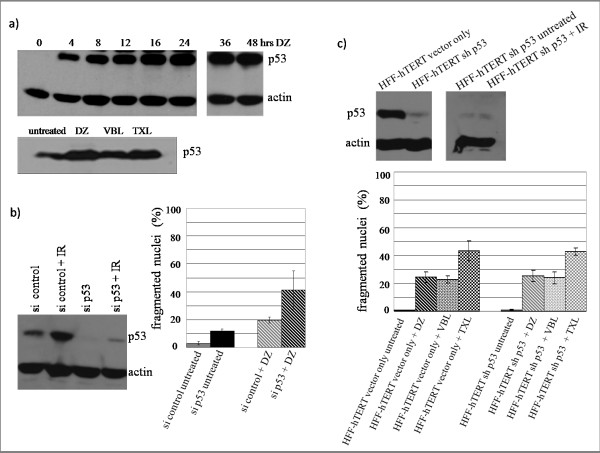
**p53 is stabilized after DZ treatment, but is not required for mitotic slippage**. IR: ionizing radiation, commonly used for inducing p53 in p53 knockdown cells to detect the efficiency of the siRNA knockdown. a) Immunoblot analyses of p53 in RPE-hTERT cells after indicated treatment. Upper panel: cells were treated with 10 nM of DZ for indicated time. Lower panel: cells were treated with 10 nM of DZ, 100 nM of VBL or 1 μM of TXL for 38 hours. b) Left panel: immunoblot analysis of p53 in RPE-hTERT p53-wild type and p53-knockdown cells. Right panel: percentages of fragmented nuclei in RPE-hTERT p53-wild type and p53-knockdown cells treated with 10 nM of DZ for 38 hours. Graph shows the average of three independent experiments. c) Upper panel: immunoblot analysis of p53 in HFF-hTERT vector only and HFF-hTERT shp53 (stable-knockdown) cells. Lower panel: percentages of fragmented nuclei in HFF-hTERT vector only and HFF-hTERT shp53 cells treated with 10 nM of DZ, 100 nM of VBL or 1 μM of TXL for 38 hours. Results in graph are averages of three independent experiments.

Elevated levels of p53 have been observed previously in cells treated with microtubule inhibitors [17], and therefore their role in mitotic slippage was examined here. As demonstrated in Figure 5a, p53 accumulated in RPE-hTERT cells after DZ treatment, comparable to that seen with VBL or TXL treatment. However, the activation of p53 was not essential for mitotic slippage, as siRNA knockdown of p53 in RPE-hTERT cells did not diminish the frequency of fragmented nuclei (Figure 5b). To further confirm this, we used human foreskin fibroblasts transformed with a plasmid expressing human telomerase with wild-type p53 (HFF-hTERT vector only) or a stable-knockdown of p53 (HFF-hTERT shp53; a gift from Dr. Edward Prochownik, University of Pittsburgh). No reduction of mitotic slippage was observed in cells unable to express p53, thus confirming that p53 was not required (Figure 5c).

### 5. Non-cancer cells undergo mitotic slippage after DZ treatment without activating apoptotic pathways, while cancer cells execute apoptosis

**Figure 6:**
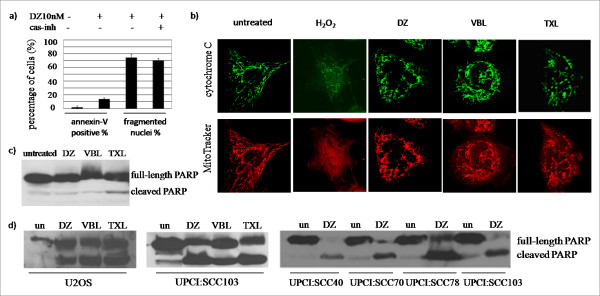
**Non-cancer cells undergo mitotic slippage without activating apoptotic pathways while cancer cells execute apoptosis after DZ treatment**. Cells were treated with 10 nM of DZ for 38 hours in the following experiments unless where indicated. 100 nM of VBL and 1 μM of TXL were used as controls. RPE-hTERT cells were used in (a-c). a) cas-inh: Caspase inhibitors (caspase-3/7, -8, and -9 inhibitors). Results are averages of three independent experiments. b) Upper panel: cytochrome C staining. Lower panel: MitoTracker staining. c) Immunoblot analysis of PARP. Upper band: full-length PARP. Lower band: cleaved product of PARP. d) Immunoblot analysis of PARP cleavage in cancer cells after DZ treatment. un: untreated.

Although mitotic slippage was previously considered a survival pathway to protect cells from apoptosis [18], emerging studies have shown that mitotic slippage can also be coupled to apoptosis [19-21]. To study whether apoptosis was responsible for the mitotic slippage from DZ exposure, we preformed four tests. Only 15% of the RPE-hTERT cells were annexin-V positive, suggesting far fewer cells underwent apoptosis than nuclear fragmentation (Figure 2b, d). Furthermore, the percentages of nuclear fragmentation did not decrease when caspase inhibitors were used (Figure 6a), indicating that mitotic slippage was independent of apoptosis. Additionally, other apoptotic markers were also analyzed. These markers include: 1) cytochrome C staining, demonstrating little release of cytochrome C after DZ treatment compared to the H_2_O_2 _control (Figure 6b, Additional file 4); 2) mitochondrial membrane potential indicator MitoTracker, revealing little loss of mitochondrial membrane potential upon DZ treatment compared to the H_2_O_2 _control (Figure 6b); and 3) poly(ADP-ribose) polymerase (PARP) cleavage, also revealing little or no apoptosis (Figure 6c).

Even when cells were treated with DZ for 68 or 72 hours, very little apoptosis was observed (data not shown). Furthermore, other non-cancer cell lines, such as HFF-hTERT (immortalized human foreskin fibroblasts) and UP3 cells (primary human oral cells grown in culture), also showed very little or no PARP cleavage after 38 hours of 10 nM DZ treatment (Additional file 5). Additionally, DNA ladder formation was not observed in RPE-hTERT cells exposed to DZ (Additional file 6). Taken together, our results indicate that the apoptotic pathway was not activated in the RPE-hTERT cells exposed to DZ. However, PARP cleavage was strongly observed in all the tested cancer cell lines (Figure 6d), suggesting apoptosis pathway was activated in malignant cells by DZ.

## Discussion

DZ is a naturally existing compound that has been recently synthesized to evaluate its potential as a cancer therapeutic. In our study, DZ treatment demonstrated a disruption of microtubules from the plus end, consistent with its proven ability to bind tubulin [12], indicating that the mechanism of action of DZ is similar to that of other classical microtubule disruptors. However, DZ treatment seemed to show stronger mitotic slippage than VBL in RPE-hTERT cells (Figure 2d), suggesting that DZ can act differently than some other microtubule destabilizers.

Previous studies of microtubule inhibitors' effects on non-transformed cells have been limited to a DNA content-based analysis of cell cycle distribution. Researchers have observed an accumulation of cells with a 4N DNA content in the second cell cycle following mitosis, referred to as mitotic slippage [22]. However, the cause of this phenotype is not fully understood. Recently, Brito and Rieder showed that mitotic slippage after nocodazole treatment occurs when the spindle-assembly checkpoint (SAC) fails to prevent a slow but continuous proteolysis of cyclin B [16]. In the current study, we observed that mitotic slippage after DZ treatment was also correlated with cyclin B degradation.

p53 has been demonstrated to play a major role in cell cycle control [23]; thus it may also be involved in mitotic slippage. In the p53-knockdown RPE-hTERT cells there was no loss of fragmentation; indicating that p53 was not required for mitotic slippage in the presence of this microtubule inhibitor. In fact, there was a consistent *increase *in mitotic slippage in the transient p53 knockdown cells. This could be due to p53 down-regulation of cdc20 [24], which is demonstrated to be required for the proteolysis of cyclin B by activating anaphase-promoting complex, a mitotic ubiquitin ligase [25]. By knocking down p53, cdc20 levels may increase, thus activating more ubiquitin ligase complexes and promoting cyclin B degradation. Alternatively, the observed increase in the percentage of cells undergoing mitotic slippage may result from cells proliferating faster after p53 knockdown, which can increase the frequency of cells passing through mitosis. However, we did not see an increase in mitotic slippage with the stable p53 knockdown and this relationship will thus require further study.

**Figure 7:**
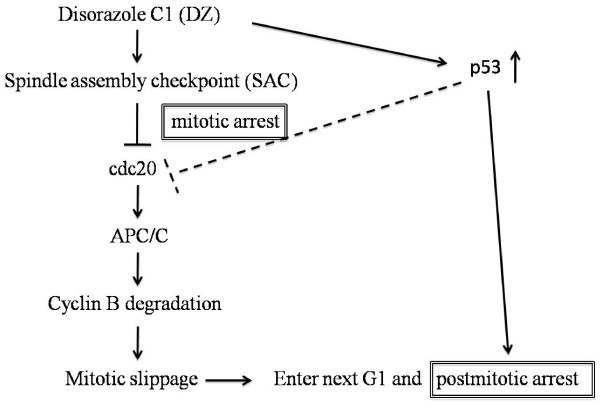
**Model: response of non-cancer cells to microtubule inhibitors**. Disorazole is proposed to activate two sequential cell cycle checkpoints. The first is the APC-dependent arrest at mitosis; when that fails, a second arrest at G1 is observed. p53 stabilization may mediate both events but is not required for the mitotic slippage that acts independent of the traditional p53-dependent apoptosis pathways. APC/C: anaphase-promoting complex/cyclosome.

It has been demonstrated that after mitotic slippage, non-cancer cells can be arrested in the next G1 stage in a p53-dependent manner [15,22]. Similarly, after DZ treatment, we also observed an accumulation of cells with a 4N DNA content (Figure 2e), as well as an induction of p53 (Figure 5a), which is similar to the postmitotic G1 arrest of TXL-treated non-cancer cells [15]. Hence, we suggest that when non-cancer cells are treated with microtubule inhibitors, for example DZ, they may have two "checkpoints" sequentially to ensure the fidelity of cell division. The first one is SAC, which can cause mitotic arrest if microtubules are disrupted [25]. If SAC is not effective enough, cells may slip out from mitotic arrest and become arrested in the next G1 stage [15,16,22]. Based on our data and previous studies, it is plausible that p53 facilitates both of these "checkpoints" after microtubule disruption as described in the model (Figure 7).

p53 has often been found compromised in human cancer tissues [26]. In our study, p53 is either truncated (UPCI:SCC103) or undetectable (HeLa) in cancer cell lines (data not shown). This indicates that the apoptosis triggered by DZ in cancer cells may be p53-independent.

Finally, our data suggest that after exposure to a certain concentration of DZ (10 nM in this study), cancer cells tend to execute apoptosis while non-cancer cells undergo mitotic slippage without activating the apoptotic pathways, indicating that non-cancer and cancer cells respond differently after DZ treatment. Those cells that do not die from apoptosis may become senescent, a phenotype discussed previously [[12] and references within].

## Conclusions

In this study, we analyzed the cellular response to a synthesized natural product, DZ. Although mitotic arrest induced by DZ was observed in both non-cancer and cancer cells, these cells had different fates. Non-cancer cells escaped from mitotic arrest (mitotic slippage) in a cyclin B-dependent and p53-independent manner without apoptosis. In contrast, cancer cells activated the apoptosis pathway. These differential responses observed after DZ treatment were similar to the effects of vinblastine or taxol, suggesting a promising therapeutic potential of DZ.

## Methods

### Cell cultures and treatments

HFF-hTERT vector only (human foreskin fibroblast immortalized with human telomerase reverse transcriptase) and HFF-hTERT shp53 (stable-knockdown of p53) cells (gifts from Dr. Edward Prochownik, University of Pittsburgh) were maintained in DMEM supplemented with 10% fetal bovine serum (FBS) and 1% L-glutamine. RPE-HTERT cells (retinal pigmented epithelial cell line stably transfected with human telomerase reverse transcriptase) were maintained in DMEM/F12 supplemented with 10% FBS. All UPCI:SCC cells (oral squamous carcinomas cells, gifts from Dr. Susanne Gollin, University of Pittsburgh) were maintained in MEM supplemented with 10% FBS and 1% non-essential amino acid. UP3 (uvulopalatopharyngoplasty) cells (gift from Dr. Susanne Gollin) were maintained in KGM2 medium. U2OS cells (ATCC) were maintained in McCoy's 5A supplemented with 10% FBS. Generally, cells were treated with 10 nM DZ or 100 nM vinblastine or 1 μM taxol for 38 hours at 37°C before analysis, unless where indicated. Vinblastine concentration was chosen to demonstrate a similar level of microtubule disruption compared with 10 nM of DZ. Cell permeable caspase inhibitors (caspase-3/7, -8, -9 inhibitors) (Calbiochem) were used as in reference Jennings JJ *et al*., 2006 [27].

### Immunofluorescence

Cells were fixed in 3.7% paraformaldehyde at room temperature for 15 minutes followed by 7 minute-incubation of 0.1% triton X-100 at 4°C. Primary α-tubulin antibodies (Abcam, Cambridge, MA) or cytochrome C antibodies (Santa Cruz) were detected with the application of Alexa Fluor488 or 568 (Molecular Probes, Invitrogen) as the secondary antibodies. All antibodies were diluted in 1% bovine serum albumin (BSA)/PBS. DNA-specific fluorescent dye 4,6-diamidino-2-phenylindole (DAPI; Sigma, St. Louis) was used to visualize DNA. The samples were examined using a 40× or 100× oil immersion objective (UPlanApo) at an Olympus BX60 epifluorescence microscope. Images were taken using a Hamamatsu Argus-20 CCD camera (Hamamatsu Co., Bridgewater, NJ)

### Live cell imaging

Cells were seeded on 35-mm glass-bottom culture dish (MatTek Corporation, Ashland, MA) and treated with DZ at a final concentration of 10 nM for 16 hours before analysis. Epifluorescence microscopy was performed on a Nikon TE2000-U inverted microscope with a CoolSNAP HQ digital camera (Roper Scientific Photometrics) while cells were maintained at 37°C in a moisturized and air-heated microscope chamber (Life Imaging Services, Reinach, Switzerland). Images were taken and analyzed using MetaMorph software (Molecular Devices).

### Western Blotting

Antibodies against cyclin B (BD, San Jose, CA), actin (Cytoskeleton, Denver, CO), p53 (Santa Cruz Biotechnology, Santa Cruz, CA), PARP (Cell Signaling Technology, Danvers, MA) were used as primary antibodies and all diluted in 5% milk/Tris-buffered Saline with 0.5% Tween-20 (TBST). Anti-mouse or anti-rabbit IgG-HRP-linked secondary antibodies (Amersham, GE Healthcare, UK) were also diluted in 5% milk/TBST. Results were visualized using enhanced chemiluminescent kit (Pierce).

### Cell proliferation and apoptosis assays

Cell proliferation was measured using the MTS assay kit (Promega; tetrazolium compound [3-(4,5-dimethylthiazol-2-yl)-5-(3-carboxymethoxyphenyl)-2-(4-sulfophenyl)-2H-tetrazolium, inner salt; MTS]) following the manufacture's instruction. In annexin-V assays, Tetramethyl Rhodamine Methyl Ester (TMRM) diluted in regular buffer was used to stain mitochondria at 37°C for 10 minutes before the addition of annexin-V (Molecular Probes, Invitrogen, CA) following the manufacture's instruction. MitoTracker (Invitrogen, CA) staining was performed following the manufacture's protocol.

### Flow cytometry

Cells were fixed with cold 70% EtOH and stained with propidium iodide (Fluka: BioChemika, Switzerland) at a final concentration of 50 μg/ml. Analysis of flow cytometry was performed in Flow Cytometry Facility in University of Pittsburgh Cancer Institute (UPCI).

### p53 knockdown

Cells were seeded onto 60-mm culture dishes five hour before siRNA transfection. sip53 (Qiagen) was transfected into cells using HiPerFect Transfection Reagent (Qiagen) following the manufacture's instruction.

### Electron microscopy

Cells grown on plastic dishes were fixed in 2.5% glutaraldehyde or paraformaldehyde in 0.1 M Na-cacodylate (30 min), washed with 0.1 M Na-cacodylate, post-fixed with 1% OsO4, washed with PBS and stained for 30 min with 2% uranyl acetate. Following dehydration in 30% to 100% ethanol, the samples were embedded in resin by immersion in 30% to 100% resin:propylene oxide mixtures. Fixed samples were mounted on grids and analyzed with a JEOL 100CX transmission electron microscope.

## Authors' contributions

FLX designed and carried out most of the experiments (except EM), and analyzed the data and drafted the manuscript. YR performed EM analysis experiments. KK participated in the design of the study and helped in the EM and microscopy experiments. JSL and PW provided DZ and advices for the study. WSS provided funding and supervision of the study and participated in designing experiments and drafting the manuscript. All authors read and approved the final manuscript.

## Supplementary Material

Additional file 1 **Figure S1**. Structures of disorazole A_1 _and DZ. Click here for file

Additional file 2 **Figure S2**. Proliferation assay of RPE-hTERT cells treated with DZ and VBL, indicating that DZ inhibited growth of RPE-hTERT cells. Click here for file

Additional file 3 **Supplemental Movie**. Cells were treated with 10 nM of DZ for 16 hours before imaging. Cells escaped from mitotic arrest and formed abnormally shaped nuclei, indicating mitotic slippage had occurred. Click here for file

Additional file 4 **Figure S3**. H_2_O_2_-treated cells demonstrating cytochrome C release retained normal microtubule structures. Left panel: α-tubulin staining. Right panel: cytochrome C staining. Click here for file

Additional file 5 **Figure S4**. Only little or no PARP cleavage was observed in UP3 (primary cells) and HFF-hTERT (non-cancer cells) after DZ treatment. un: untreated. Click here for file

Additional file 6 **Figure S5**. DNA fragmentation assay using untreated REP-hTERT cells and RPE-hTERT cells treated with DZ revealed little DNA laddering upon DZ treatment (left panel) while H_2_O_2_-treated RPE-hTERT cells demonstrated DNA laddering (right panel). An arrow indicates DNA ladder, a marker of apoptosis. Click here for file
