# Supplementary figures and images for: Mitotic slippage in non-cancer cells induced by a microtubule disruptor, disorazole C1
Source: BMC Chem Biol. 2010 Feb 11;10:1. doi: 10.1186/1472-6769-10-1 (PMC2834648; doi:10.1186/1472-6769-10-1)

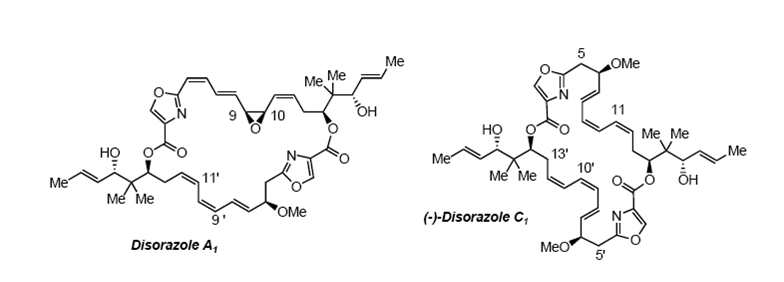

Supplement: Additional file 1 — Figure S1. Structures of disorazole A1 and DZ. [file 1472-6769-10-1-S1.PNG]

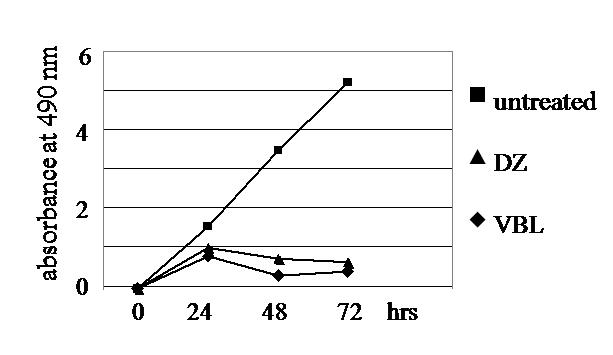

Supplement: Additional file 2 — Figure S2. Proliferation assay of RPE-hTERT cells treated with DZ and VBL, indicating that DZ inhibited growth of RPE-hTERT cells. [file 1472-6769-10-1-S2.PNG]

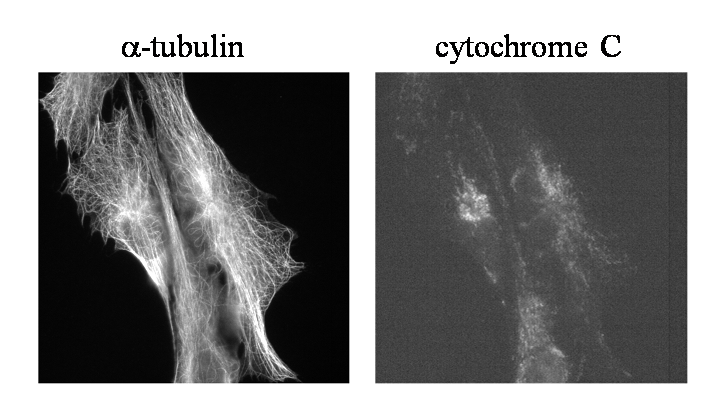

Supplement: Additional file 4 — Figure S3. H2O2-treated cells demonstrating cytochrome C release retained normal microtubule structures. Left panel: α-tubulin staining. Right panel: cytochrome C staining. [file 1472-6769-10-1-S4.PNG]

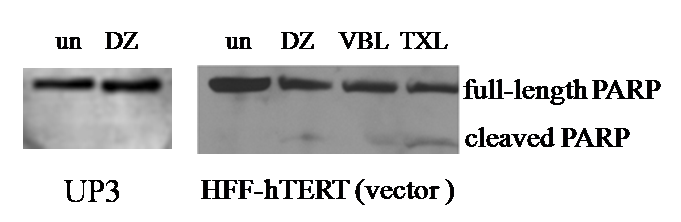

Supplement: Additional file 5 — Figure S4. Only little or no PARP cleavage was observed in UP3 (primary cells) and HFF-hTERT (non-cancer cells) after DZ treatment. un: untreated. [file 1472-6769-10-1-S5.PNG]

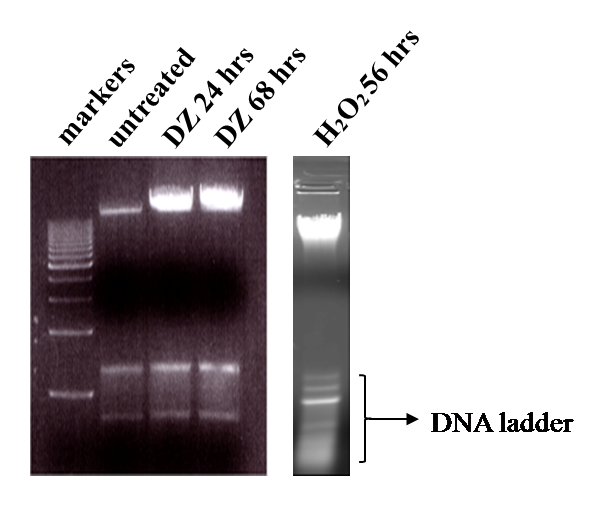

Supplement: Additional file 6 — Figure S5. DNA fragmentation assay using untreated REP-hTERT cells and RPE-hTERT cells treated with DZ revealed little DNA laddering upon DZ treatment (left panel) while H2O2-treated RPE-hTERT cells demonstrated DNA laddering (right panel). An arrow indicates DNA ladder, a marker of apoptosis. [file 1472-6769-10-1-S6.PNG]
